# Recurrent Pericardial Effusion Secondary to Cardiac Angiosarcoma

**DOI:** 10.7759/cureus.60460

**Published:** 2024-05-16

**Authors:** Zahid Khan

**Affiliations:** 1 Acute Medicine, Mid and South Essex NHS Foundation Trust, Southend-on-Sea, GBR; 2 Cardiology, Bart’s Heart Centre, London, GBR; 3 Cardiology and General Medicine, Barking, Havering and Redbridge University Hospitals NHS Trust, London, GBR; 4 Cardiology, Royal Free Hospital, London, GBR

**Keywords:** pericardial tamponade, pericardial effusion. cardiac tamponade, autoimmune screening, pericardial fluid analysis, cardiac magnetic resonance (cmr), emergency echocardiography, palliative chemotherapy, recurrent pericardial effusion, primary cardiac angiosarcoma, primary cardiac tumours

## Abstract

Cardiac angiosarcoma is a malignant cardiac tumour. We present the case of a young patient in his mid-30s with recurrent pericardial effusion. He had flu-like symptoms a month earlier and had shortness of breath, lethargy, and tightness in his throat for the past ten days. Echocardiography demonstrated global pericardial effusion > 4 cm with tamponade features, and the patient was blue-lighted to our hospital. He underwent emergency pericardiocentesis, and > 1 litre of pericardial fluid was drained. Computed tomography of the chest, abdomen, and pelvis revealed small-volume ascites and moderate right-sided pleural effusion, with associated lobar collapse. The patient presented to the hospital with global pericardial effusion requiring emergency pericardiocentesis three weeks later and underwent cardiac magnetic resonance imaging demonstrating global pericardial effusion and a 48 × 26 mm pericardial space mass adjacent to the right atrium. He underwent surgical resection of the tumour, followed by chemotherapy, and tolerated the treatment well. The patient is currently under follow-up.

## Introduction

Primary cardiac tumours are very rare, with approximately 0.002%-0.33% detected in autopsies while the incidence of malignant cardiac tumours is 0.0017%-0.28% [1,2]. Approximately 75% of cardiac tumours are benign, including rhabdomyoma, cardiac myxoma, hemangioma, fibroma, and teratoma, with cardiac myxoma being the most common [2]. Malignant tumours, such as lymphoma, sarcoma, and mesothelioma, account for 25% of cardiac tumours, with angiosarcoma being the most common [1]. Cardiac angiosarcomas arise from the right side of the heart, and 89% of malignant cardiac tumours metastasize at the time of diagnosis. Angiosarcomas originate from endothelial cells and commonly occur in men 30-50 years old and account for 28.6% of malignant cardiac tumours [2-4]. Approximately 90% of cardiac angiosarcomas originate from the lateral wall and are in the right atrium (RA). The left atrium is the second most common location for cardiac angiosarcomas, followed by the right and left ventricles [1]. Patients with cardiac angiosarcomas have a poor prognosis owing to late detection, challenging anatomy, and incomplete tumour resection, with a median survival of 14 months in surgically treated patients [1,2]. The clinical presentation varies depending on the infiltration of the myocardium and the extent of metastases, and these patients may present with chest pain, shortness of breath, congestive cardiac failure, arrhythmia, and pericardial tamponade [5]. We present a case of a young patient with recurrent pericardial effusion who required repeated pericardiocentesis secondary to cardiac angiosarcoma.

## Case presentation

A patient in his 30s presented with a two-day history of palpitations, chest discomfort, and shortness of breath. He described his symptoms to be like those of his previous episode about a month ago when he was admitted with a large pericardial effusion causing cardiac tamponade and requiring emergency pericardiocentesis. He denied any coryzal symptoms. He was tachycardic and hypotensive when he presented to the Accident and Emergency department. His medical history included a recent chest infection and emergency pericardiocentesis for cardiac tamponade a month ago. Computed tomography (CT) of the chest, abdomen, and pelvis during the last admission revealed a right-sided moderate pleural effusion with dense left lower lobe consolidation and a small volume of ascites with no evidence of malignancy. The patient received a seven-day course of intravenous amoxicillin and clavulanic 1.2 gram initially, followed by a four-day oral course following pericardiocentesis, and showed complete recovery for a short time. Pericardial fluid microbiology and cytology analyses were negative for any microbacteria, acid-fast bacilli, and malignancy. Biochemical analysis revealed that pericardial effusion was exudative. He was not on any regular medications and did not smoke or drink alcohol.

**Figure 1:**
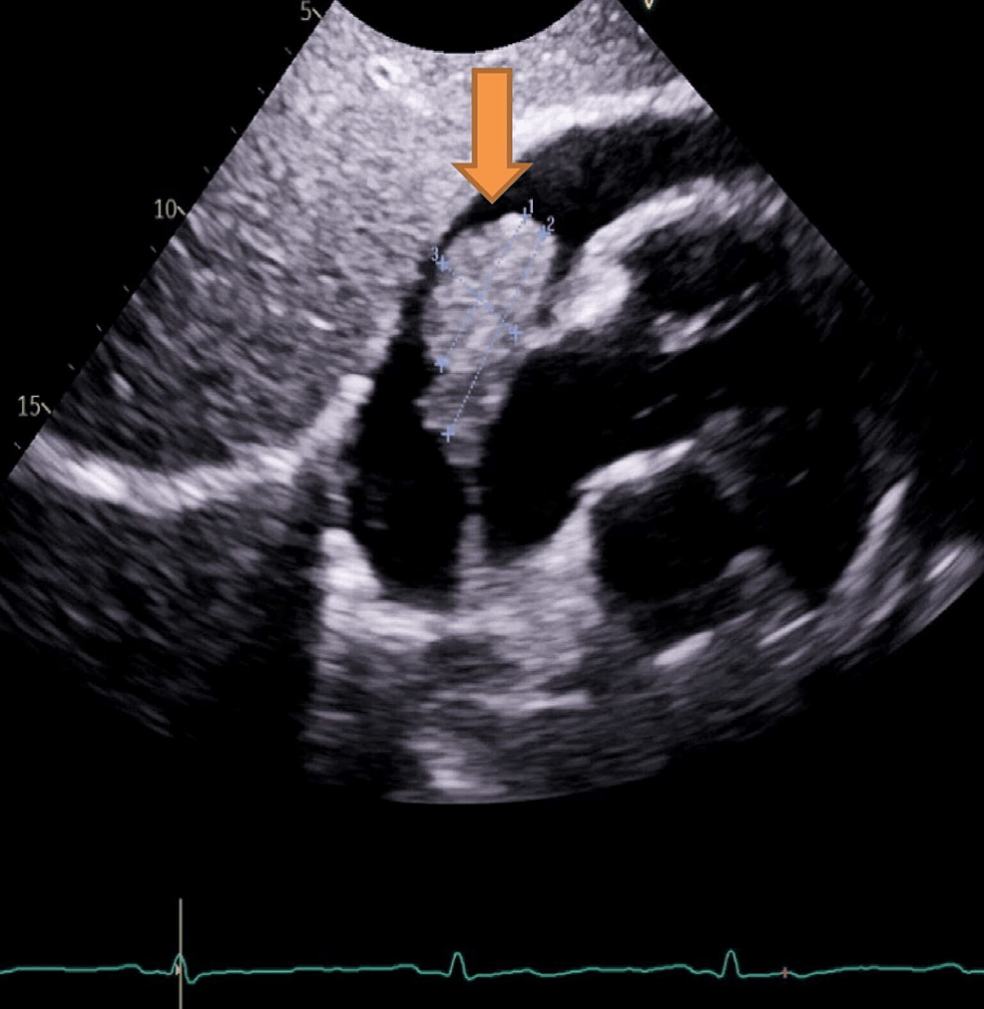
Echocardiography subcostal view showing large pericardial effusion and a right atrial mass (coloured arrow)

**Figure 2:**
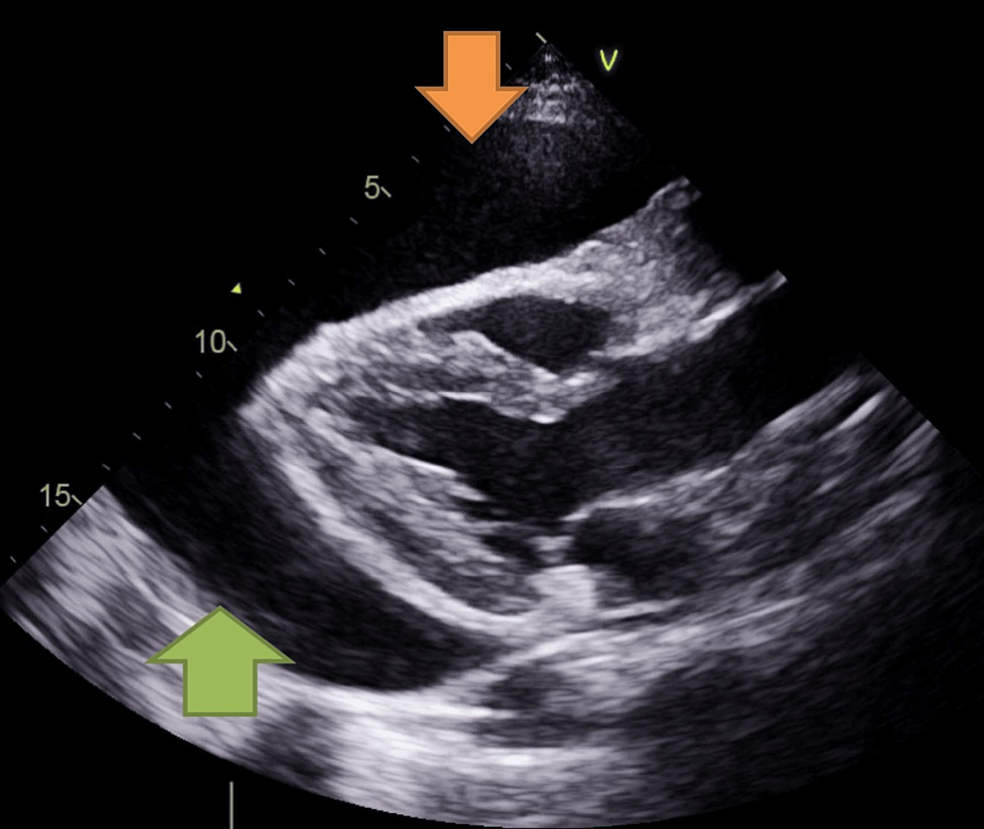
Echocardiography parasternal long-axis view showing large pericardial effusion (pointed coloured arrows)

**Figure 3:**
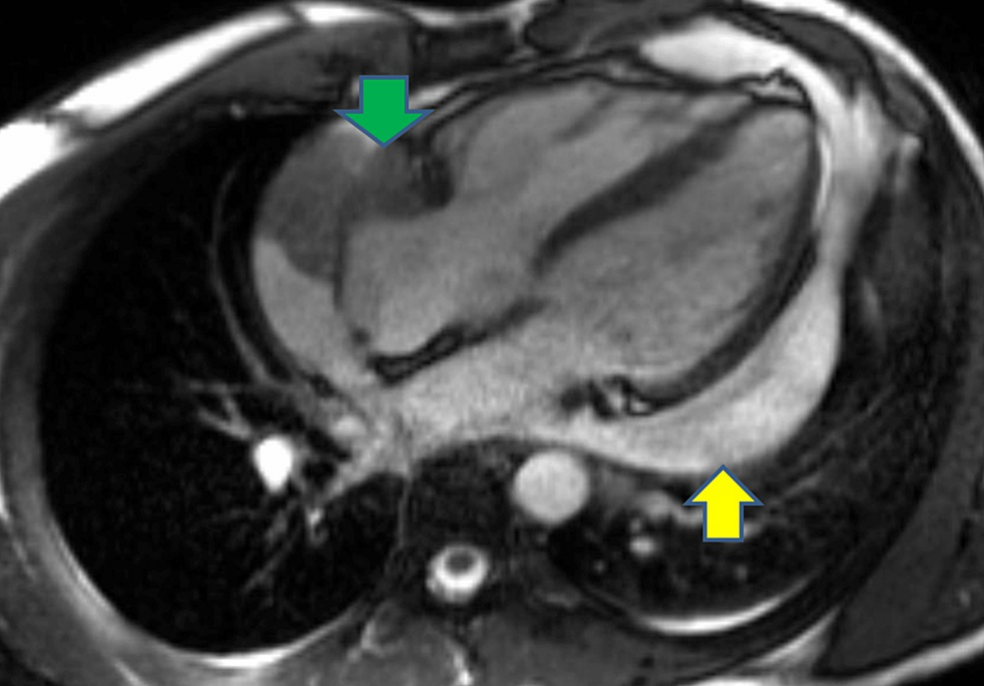
Cardiac magnetic resonance imaging showing pericardial effusion (yellow arrow) and right atrial mass (green arrow)

On clinical examination, his chest was clear with normal heart sounds. Electrocardiography showed sinus tachycardia with a heart rate of 108 beats per minute. His vital signs were as follows: blood pressure, 97/81 mmHg; heart rate, 108 beats per minute; oxygen saturation (SpO_2_ 95% on room air); respiratory rate, 17; and apyrexial. Chest radiography revealed cardiac enlargement without any obvious consolidation. Echocardiography demonstrated global pericardial effusion measuring 2.2 cm around the right ventricle, 2.4 cm around the right atrium, 2.5 cm around the left ventricular lateral wall, 1.35 cm around the right ventricular apex, with normal biventricular function (Figures 1-2). Autoimmune screening results were negative, and laboratory test results showed anaemia and elevated levels of inflammatory markers (Table 1). Echocardiography also demonstrated a 4 x 2.2 cm echogenic mass attached to the right atrial wall in most views (Figure 1). The patient was administered 250 mL of normal saline for hypotension and was urgently transferred to our tertiary centre. He was discussed by a cardiothoracic team for the pericardial window given the recurrent pericardial effusion, who advised the pericardial window at that point. He underwent cardiac magnetic resonance imaging (MRI), which revealed an irregular mass in the pericardial space infiltrating the right atrium, with a vascular appearance based on early gadolinium and perfusion kinetics (Figure 3). The left ventricular function was mildly impaired on cardiac MRI without any evidence of late gadolinium enhancement.

**Table 1:** Laboratory test results for patients during admission

Lab Test	Day 1	Day 5	Reference Values
Haemoglobin	84	96	120-150 g/L
White cell count	5.6	6.6	4-10 x10^9/L
Platelets	177	377	150-410 x10^9/L
Neutrophil	5.6	3.9	2-7 x10^9/L
Mean cell volume	80.7	82.3	83-101 fl
Urea	2.0	2.3	2.5-7.8 mmol/L
Creatinine	70	77	45-84 umol/L
Sodium	143	141	133-146 mmol/L
Potassium	4.1	4.5	3.5-5.3 mmol/L
C-reactive protein	204	99	0-5 mg/L
Erythrocyte sedimentation rate (ESR)	13	18	0-13 mm/hour

The patient was reviewed by the cardiothoracic team, and surgical resection of the tumour was recommended by the cardiothoracic multidisciplinary team meeting (MDT). The patient underwent open median sternotomy for surgical resection of the mass and a pericardial window to drain pericardial effusion, as the patient had a significant amount of bleeding during the procedure requiring two units each of fresh frozen plasma, prothrombin complex concentrate, platelets, and cryoprecipitate. The patient had a period of instability during anesthesia induction due to hypotension and a bleeding mass over the right atrium invading the pericardium and pleura on opening the chest wall. The mass was about 6 cm in diameter with soft and hard components and inseparable from the right atrium and pleura. Hence, partial resection of the mass from both the right atrium and pleura was performed. The raw bleeding areas were sutured, and mediastinal, pericardial, and right pleural drains were inserted. The surgery took almost 2.5 hours from the start of the induction of general anaesthesia. The patient was admitted to the intensive care unit postoperatively and the drains were removed after 48 hours (about 2 days) of monitoring. He stayed in ICU for 3 days followed by a further 3 days stay in the cardiothoracic ward. Histology revealed a malignant tumour composed of pleomorphic ovoid tumour cells with cytological atypia and mitotic activity. There were areas of necrosis and anastomosing vascular channels lined by plumps, pleomorphic cells with multi-layering, and extravasated red blood cells. The morphological and immunohistochemical findings were consistent with those of angiosarcoma. Repeat echocardiography on postoperative day 3 showed a small pericardial effusion approximately 0.7 cm behind the right atrium and left ventricle with no evidence of chamber collapse.

He was referred to another centre to commence palliative chemotherapy for stage 3 angiosarcoma and has completed three cycles of taxanes (paclitaxel and docetaxel) to date. Repeat echocardiography two months later showed a very small pericardial effusion (0.5 cm). He tolerated chemotherapy well, and his sternotomy wound had completely healed.

Outcome and follow-up****

The patient completed three cycles of chemotherapy, and he remains under follow-up with the oncology and cardiothoracic teams. Chemotherapy was tolerated without major side effects. Repeat echocardiography revealed a mild pericardial effusion.

Differential diagnosis****

The most common differential diagnosis includes non-cardiac metastatic malignancy ruled out by negative cytology and CT of the chest, abdomen, and pelvis. Tuberculosis was ruled out using a negative acid-fast bacilli test and CT scan. Tests for autoimmune diseases, such as systematic lupus erythematosus and rheumatoid arthritis, were negative. Infections such as bacterial and viral infections can also cause recurrent pericardial effusion; however, this patient did not show any evidence of infection on his second admission, although he was treated for lobar pneumonia during his first admission. Myocardial infarction and heart failure can present with pericardial effusion; however, this patient did not show any evidence of myocardial infarction, and his left ventricular ejection fraction was only mildly impaired on echocardiography and cardiac magnetic resonance imaging. Cardiac tumours, such as leiomyomas, rhabdosarcoma, and teratomas, can also present with recurrent pericardial effusion; however, these were ruled out based on the histological findings from the biopsy.

## Discussion

Primary cardiac malignancy is rare, with a reported incidence of approximately 25% [5]. These tumours usually metastasize at the time of diagnosis and commonly involve the lung (20%-55%), liver (10%-22%), and bone (10%-20%) [5]. Patients usually present with atypical symptoms such as dyspnea, chest pain, and pericardial effusion. Primary cardiac angiosarcoma is the most aggressive type of malignant primary cardiac tumour, accounting for approximately 33% of all primary cardiac malignant tumours [5,6]. Patients with malignant cardiac tumours have local and distant metastases at the time of diagnosis, and conservative management or palliative chemotherapy is possible in most cases. The poor prognosis of these tumours is due to delayed diagnosis, tumour metastasis, various arrhythmias, and refractory heart failure [6]. Cardiac sarcomas predominately occur in males aged 30-50 years and most commonly involve the right side of the heart. Patients most commonly present with symptoms and signs of right-sided heart failure [7]. The most common presenting signs and symptoms include dyspnea, pericardial effusion, palpitations, chest pain, and myalgia. Angiosarcoma rarely presents with cardiac tamponade because of significant pericardial effusion [7].

The prognosis for angiosarcoma is poor [7]. A case report by Kennedy et al. described a 32-year-old patient with recurrent pericardial effusion and pleural effusion requiring pericardial window, who was diagnosed with angiosarcoma [7]. The patient needed two pericardial windows over six months for recurrent pericardial effusions and a chest drain for pleural effusion. Computed tomography of the chest, abdomen, and pelvis revealed a mediastinal tumour compressing the right ventricle and atrium, which was confirmed using transthoracic echocardiography. Pleural fluid cytometry and cytology demonstrated clusters of neoplastic cells, and flow cytometric analysis showed CD45 negative cells that lacked BerEP4 and E-cadherin expression, consistent with vascular neoplasms such as angiosarcoma. Primary cardiac angiosarcoma can also be misdiagnosed because of its rarity [8]. Zaheer et al. published a case report of a 46-year-old male who underwent computed tomography and transesophageal echocardiography for chest pain, dyspnea, and intermittent fever that demonstrated a right atrial pseudoaneurysm. The patient underwent resection of the pseudoaneurysm due to concerns about a possible rupture, and the histopathology of the resected pericardial mass was positive for angiosarcoma [8]. The early diagnosis of this cancer is crucial because of its aggressive nature and potential for metastasis.

There is no standardized approach to the management of these tumours because of their rarity and high mortality rate [8,9]. The average survival without surgical resection is 4 months, and the mainstay of treatment is surgical resection of the tumour, with a postoperative median survival of approximately 14 months [8]. It is important to mention that complete resection of tumours can be extremely challenging due to delayed diagnosis and proximity of the tumour to vascular structures. Chemotherapy and radiotherapy are used as adjuncts or neoadjuvant therapies; however, their exact roles are unclear [8,10].

Computed tomography and MRI scans are superior to echocardiography in providing a more detailed description of the cardiac soft tissues, including extracardiac involvement and metastasis. CT also provides information about the calcification of the tumour, which is an advantage over MRI. Cardiac MRI is, however, superior to CT scan for tissue characterization and for evaluating intrinsic myocardium abnormalities. Additionally, cardiac MRI can help differentiate between neoplastic diseases and thrombi through delayed gadolinium enhancement [11]. Complete surgical resection of the tumour is possible in cases without evidence of metastasis when the tumour is suitable for curative resection. Local recurrence of the tumour is common despite complete tumour resection in cases, and about one-third of patient mortality is attributed to local recurrence of the disease [11-13].

## Conclusions

Cardiac angiosarcoma is a malignant cardiac tumour associated with a poor prognosis due to delayed diagnosis. Complete surgical resection of the tumour is usually not possible owing to delayed diagnosis. Echocardiography and cardiac magnetic resonance imaging are useful in diagnosing this condition, and histopathology is the gold standard. Patients can present with various symptoms, and our patient presented with a recurrent pericardial effusion. Treatment mostly includes surgical resection where possible, along with palliative or neoadjuvant chemotherapy.

## References

[1] Fang X, Zheng S (2021). Primary cardiac angiosarcoma: a case report. J Int Med Res.

[2] Guo Y, Liu Q, Wu H (2023). Primary cardiac tumor: a case report of right atrial angiosarcoma and review of the literature. Front Oncol.

[3] Ostrowski S, Marcinkiewicz A, Kośmider A, Jaszewski R (2014). Sarcomas of the heart as a difficult interdisciplinary problem. Arch Med Sci.

[4] Jaksic Jurinjak S, Vincelj J, Rudez I (2019). Primary left atrial angiosarcoma presenting as acute coronary syndrome. Heart Surg Forum.

[5] Farzin AO, Nejad SS (2023). Cardiac angiosarcoma: a case report. J Int Med Res.

[6] Xie J, Yuan L, Qin Y, Liu J (2023). Clinical presentation, diagnosis, and management of primary cardiac tumor: a case report. Asian J Surg.

[7] Kennedy S, Dimza M, Jones D, Seifert R (2022). An innovative approach to the diagnosis of cardiac angiosarcoma. Cureus.

[8] Zaheer S, Zhou AL, Gross JM, Kilic A (2024). Unusual presentation and delayed diagnosis of cardiac angiosarcoma. J Cardiothorac Surg.

[9] Lee CH, Chan GS, Chan WM (2003). Unexplained recurrent pericardial effusion: a lethal warning?. Heart.

[10] Teoh VW, Kamarulata IL, Loch A (2023). Recurrent pericardial effusion: a treatable cause not to be overlooked. Heart.

[11] Riles E, Gupta S, Wang DD, Tobin K (2012). Primary cardiac angiosarcoma: a diagnostic challenge in a young man with recurrent pericardial effusions. Exp Clin Cardiol.

[12] Sakaguchi M, Minato N, Katayama Y, Nakashima A (2006). Cardiac angiosarcoma with right atrial perforation and cardiac tamponade. Ann Thorac Cardiovasc Surg.

[13] Burke AP, Cowan D, Virmani R (1992). Primary sarcomas of the heart. Cancer.

